# The cytology of giant solitary trichoepithelioma

**DOI:** 10.4103/0970-9371.71874

**Published:** 2010-07

**Authors:** Jayashree Krishnamurthy, KN Divya

**Affiliations:** Department of Pathology, Medical College (VIMS), Bellary, Karnataka, India

**Keywords:** Giant solitary trichoepithelioma, basal cell carcinoma, microcystic adnexal carcinoma, trichoblastoma, FNAC

## Abstract

Giant solitary trichoepithelioma (GST) is a rare trichogenic tumor, which may present as a pigmented lesion. An 80-year-old man was diagnosed to have giant solitary trichoepithelioma on fine-needle aspiration cytology. The cytological findings represented the histological features. The recognition of GST is important because of its close resemblance to basal cell carcinoma and other skin adnexal tumors – clinically, cytologically and histologically.

## Introduction

Giant solitary trichoepithelioma (GST) is a rare hair follicle tumor with mixed epithelial and mesenchymal proliferations, consisting of basaloid epithelial strands and cellular fibromyxoid stroma. These tumors have clinicopathological resemblance to basal cell carcinoma. The cytological features of a case of GST are reported here.

## Case Report

**Figure 1:**
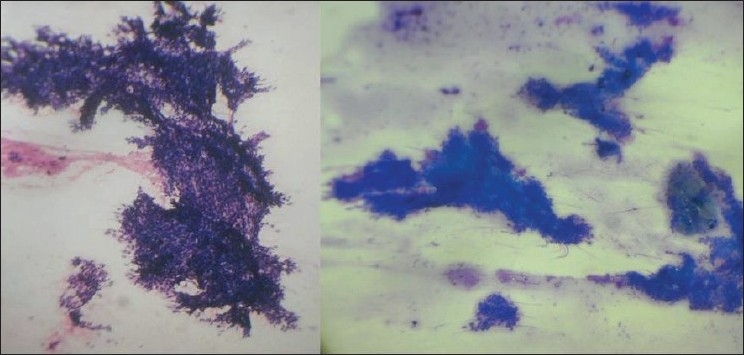
Smears showing frond-like pattern of basaloid epithelial cells(H and E; MGG, ×100)

**Figure 2:**
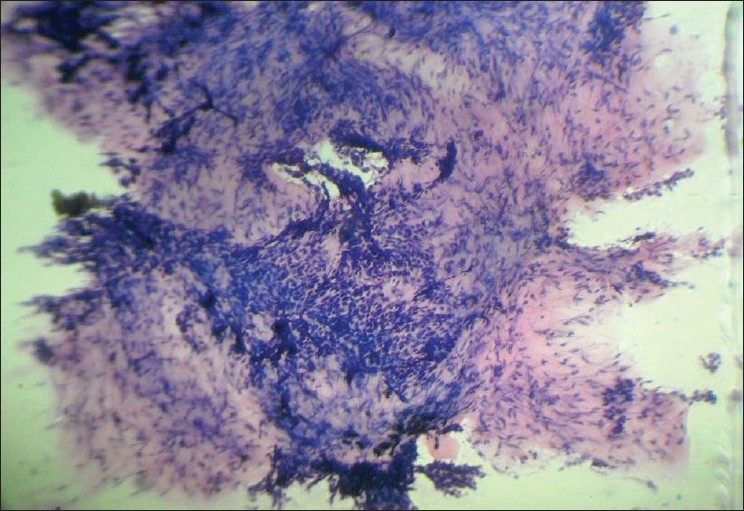
Smear showing papillary mesenchymal body (H and E, ×100)

**Figure 3:**
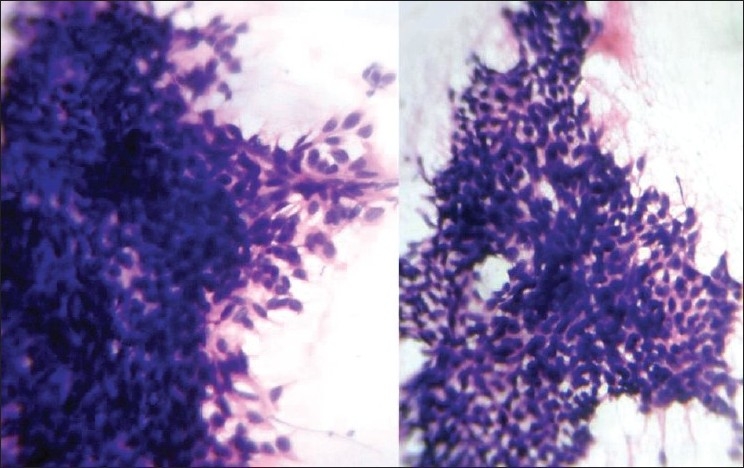
Smears showing uniform basaloid cells arranged as nests and adenoid pattern (H and E, ×400)

**Figure 4:**
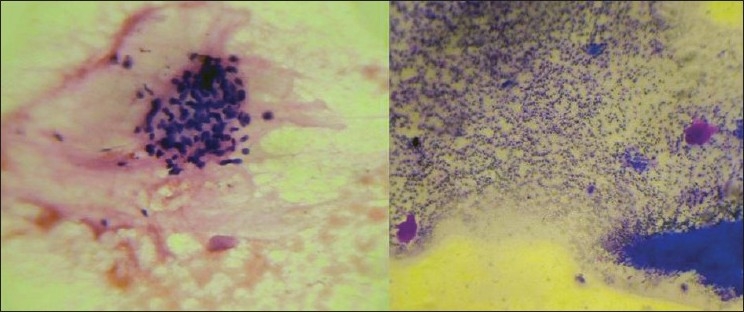
Microphotograph of FNAC smears showing abrupt keratinization (H and E, MGG ×400)

**Figure 5:**
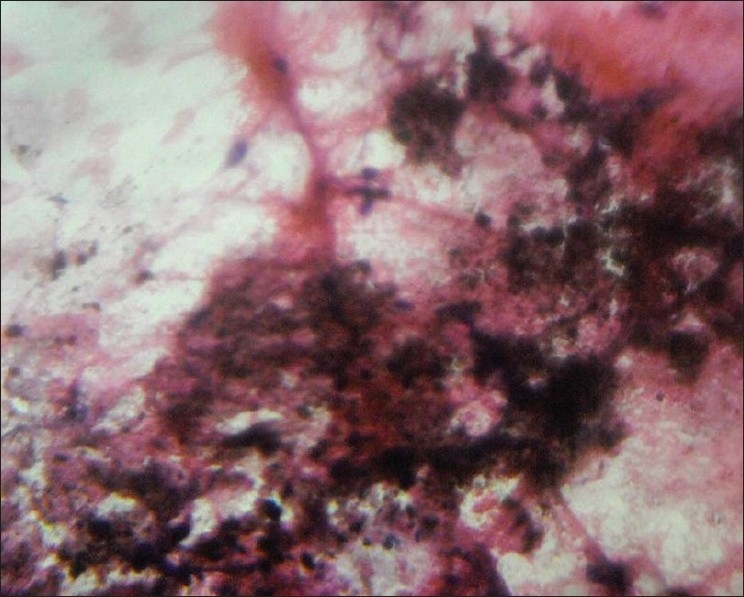
Smears showing melanin pigmentation (H and E ×400)

An 80-year-old male presented with a slowly growing pigmented swelling on the ala of the right side of the nose of 1 year duration. The local examination revealed a pigmented pedunculated swelling measuring 3×2 cm. It was firm, non-tender, immobile and was in the subcutaneous plane. The general physical and systemic examinations were normal. A clinical diagnosis of basal cell carcinoma was made. The routine investigations were normal. Fine-needle aspiration of the swelling was done and a hemorrhagic aspirate was obtained. Examination of the hematoxylin–eosin (H and E)- and May–Grünwald–Giemsa (MGG)-stained smears revealed a highly cellular aspirate consisting of frond-like pattern of basaloid cells [Figure 1] and papillary mesenchymal bodies [Figure 2]. The epithelial component consisted of uniform basaloid cells with scant cytoplasm and darkly stained nucleus arranged in nests and adenoid pattern [Figure 3]. The mesenchymal component consisted of spindle-shaped cells in a myxoid stroma. The spindle-shaped cells were also seen traversing the epithelial component. Acellular eosinophilic bodies, which represented abrupt keratinization [Figure 4], and brownish pigments [Figure 5] were documented. A cytological diagnosis of cutaneous adnexal tumor, probably trichoepithelioma, was offered.

An excision biopsy was done and was subjected to histopathological examination. Grossly the tissue was sharply demarcated, solid in consistency, white in color and lobulated in nature.

**Figure 6:**
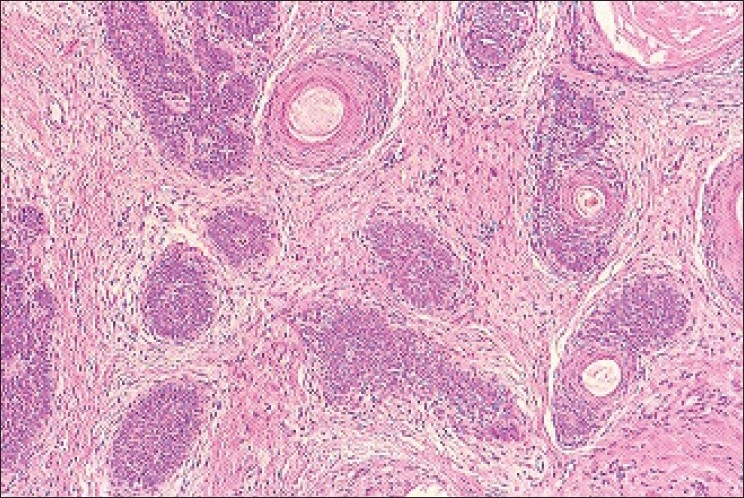
Section showing epithelial islands with follicular differentiation, fibrotic stroma is closely associated with epithelial islands, retraction artefact is absent (H and E, ×400)

Microscopy of H and E-stained paraffin sections showed a subcutaneous tumor with a normal overlying epidermis. At scanning magnification, there was a dome-shaped epithelial neoplasm within the dermis. The epithelial islands did not connect to the overlying epidermis. The tumor consisted of uniform basaloid cells arranged as anastomosing cords, adenoid pattern and epithelial islands. The epithelial cells were congregated in immature hair cell structures representing follicular differentiation. Horn cysts with fully keratinized centre surrounded by basaloid cells were observed [Figure 6]. The stroma was fibromyxoid and was closely associated with the epithelial islands. Retraction artefact as seen in basal cell carcinoma was absent in these sections [Figure 6]. These features were consistent with GST.

## Discussion

Trichoepithelioma is a trichogenic tumor described by Brooke in 1892 and later by Fordyce. It arises from the inferior segment of hair follicle epithelium.

GST is a distinct variant of trichoepithelioma. It arises in elderly individuals and occurs mostly on face, thigh and peri-anal region.[1,2] It measures several centimetres in diameter. These features are in contrast to the conventional trichoepithelioma, which presents as multiple small translucent circumscribed papules of 2–4 mm in diameter, in children and young adults. GST has a potential for local recurrence.

A cytodiagnosis of GST is suggested by frond-like epithelial component consisting of uniform basaliod cells, epithelial tracts comprising of layers of basaloid cells showing peripheral palisading and papillary mesenchymal bodies.[3] The papillary mesenchymal bodies are distinct fibroblastic aggregations that represent abortive attempts to form papillary mesenchyme responsible for hair induction.[3] Trichelemmal keratinization, which is abrupt and complete is represented by islands of basaloid cells surrounded by glassy eosinophilic areas.

GST presents as a pigmented lesion because of the increased activity of melanocytes or increased retention of pigments in the basal keratinocytes.[4] Additional findings, such as amyloidosis, inflammation, granulomas, foreign-body giant cell reactions, calcification and apoptotic bodies, are seen in giant solitary trichoepithelioma.[3,5]

The cytological differential diagnoses of GST are keratotic basal cell carcinoma, trichoblastoma and microcystic adnexal carcinoma. Keratotic basal cell carcinoma is characterized by undifferentiated basaloid cells, parakeratotic cells and trichelemmal keratinization.[6] Papillary mesenchymal bodies and uniform basaloid cells lacking atypia differentiate GST from keratotic basal cell carcinoma. Trichoblastoma (trichoblastic fibroma) is located on trunk and lacks keratinizing cysts.[7] Microcystic adnexal carcinoma, though located on the upper lip and nasolabial fold, is a poorly circumscribed invasive dermal tumor with pleomorphic ductal epithelial cells and basaloid keratinocytes.[8]

## Conclusions

GST is a rare trichogenic tumor that can be diagnosed by fine-needle aspiration cytology. It is very important to acknowledge the various cytological differential diagnoses to GST. The correct cytodiagnosis helps to outline the surgical management.

## References

[1] Tatnall FM, Jones WE (1986). Giant solitary trichoepithelioma located in the perianal area: a report of three cases. Br J Dermatol.

[2] Filho GB, Toppa NH, Miranda D, Matos MP, da Silva AL (1984). Giant solitary trichoepithelioma. Arch Dermatol.

[3] Betten Court MS, Prieto VG, Shea CR (1999). Trichoepithelioma: 19 year clinicopathologic re-evaluation. J Cutan Pathol.

[4] Rosai J (2004). Skin. Rosai and Ackerman’s surgical pathology.

[5] Gray HR, Helwig EB (1963). Epithelioma adenoides cysticum and solitary trichoepithelioma. Arch Dermatol.

[6] Kirkham N, Elder D, Elenitsas R, Jaworsky C, Johnson B (1997). Tumors and cysts of epidermis. Levers histopathology of the skin.

[7] Gilks CB, Clement PB, Woods WS (1989). Trichoblastic fibroma. Am J Dermatopathol.

[8] Goldstein DJ, Barr RJ, Santa Cruz DJ (1982). Microcystic adnexal carcinoma: a distinct clinicopathologic entity. Cancer.

